# Comparing local perspectives on women’s health with statistics on maternal mortality: an ethnobotanical study in Bénin and Gabon

**DOI:** 10.1186/1472-6882-14-113

**Published:** 2014-03-28

**Authors:** Alexandra M Towns, Tinde van Andel

**Affiliations:** 1 Naturalis Biodiversity Center, Leiden University, Darwinweg 4, P.O. Box 9517, RA 2300 Leiden, The Netherlands

**Keywords:** Gynecology, Herbal medicine, Maternal morbidity, Reproductive health, Infertility, Menstruation, Africa

## Abstract

**Background:**

According to the World Health Organization (WHO), reproductive health problems are the leading cause of morbidity and mortality for women in Africa. In spite of this scenario and the importance of plants in African health care, limited research has been conducted linking maternal health and plant-based medicine. The objective of our research was to examine how closely Beninese and Gabonese women’s health perspectives, medicinal plant knowledge, and plant use practices reflect the statistical causes of maternal mortality.

**Methods:**

In Bénin (2011) and Gabon (2012), we conducted 87 ethnobotanical questionnaires with the corresponding collection of 800 botanical specimens. We used free-listing analysis, citation frequency and species counts to determine women’s top health concerns. We also interviewed 18 biomedical healthcare providers in national hospitals and local clinics.

**Results:**

Informants’ perceptions of the main causes of maternal suffering included malaria, infertility, and menstruation and pregnancy concerns. Women were knowledgeable on plants to treat the top causes of maternal morbidity, but knew more plants for conditions such as anemia, infertility, breast milk production, and the maintenance of menstruation and pregnancy. The biomedical staff recognized the role of traditional medicine in their patients’ lives and expressed concern for herbal remedies to facilitate birth, but were restricted by national policies on advising on medicinal plant use.

**Conclusions:**

Plants serve as an entry point to understanding Beninese and Gabonese women’s perceptions of common health concerns and local health management strategies. Plant use practices in both countries did not closely parallel the top statistical causes of maternal mortality, but highlighted key issues such as menstruation and infertility as salient health concerns for women. More research is needed on the role of plants in women's gynecological healthcare.

## Background

Gynecological morbidity is among the most severe health issues in the developing world [1,2]. In Africa specifically, the major statistical causes of maternal mortality are hemorrhage (34%), sepsis/infections (10%), and hypertensive disorders (9%) [3,4]. These health conditions have contributed to the response of major international health organizations in creating the fifth goal of the United Nations Millennium Development Goals (MDGs): to improve maternal health by drastically reducing the maternal mortality ratio and achieving universal access to reproductive healthcare by 2015 [5]. Although national governments in Bénin, West Africa and Gabon, Central Africa have signed on to the MDGs, and some progress has been acquired between 1990 and 2010, neither country is on track to meet its target for 2015 [6]. In 2010, the maternal mortality ratio for Bénin was 350 per 100,000 live births and 230 per 100,000 live births in Gabon [7].

In spite of these health conditions, and widespread international and national commitment to achieving improved reproductive health, little research has been conducted on the role of medicinal plants in African women’s healthcare. This scenario is a startling contrast to the daily lives of Africans, as traditional medicine is the primary form of healthcare for 80% of the African population [8]. Even more notable is the lack of women’s knowledge in ethnobotanical research [9], in spite of the specialized knowledge women have on medicinal plants [10]. Women depend largely on traditional medicine in rural areas, where health centers are poorly equipped [11,12], but also urban areas, where biomedical treatment is offered in modern hospitals and health centers [13,14]. For over twenty years, doctors and anthropologists have expressed their concerns about the frequent use of herbs as menstrual inducers and vaginal drying agents in West Africa and Western Central Africa [15-18], yet medicinal plant use for reproductive health issues is still largely understudied [19,20].

What is missing from the current understanding of African women’s health is how African women perceive and manage their own health [21], particularly through their use of plants. Not only could documented plant use patterns identify the important plant species used in women’s health, but also the health priorities and practices of urban and rural African women. The aim of this paper was to examine how closely African women’s health perceptions, plant knowledge, and plant use practices parallel the statistical causes of maternal mortality prioritized by national governments and international organizations. Through analyzing women’s knowledge and use of medicinal plants, we sought to understand local perspectives on women’s health, considering the knowledge and use of medicinal plants to be indicators of how Beninese and Gabonese women manage their own health. We included the perceptions of local government and private healthcare providers in order to capture the local biomedical viewpoint. We posed the following questions: (1) *Among all plants used for women’s health, how many are used to treat the statistical causes of maternal morbidity and mortality?* (2) *What percentage of plants is used to treat locally-determined reproductive health concerns not addressed by international health organizations?* (3) *How do local biomedical healthcare providers perceive the use of traditional plant-based medicines for women’s health?* We expected plant use patterns to closely reflect the major maternal illnesses identified by international health organizations. Outcomes of this study can inform (inter) national health agendas in Bénin and Gabon, contribute to better understanding local medicinal practices, and serve as a starting point for further research on plant efficacy and safety with regard to maternal health.

## Methods

### Research ethics

The research team worked according to the Code of Ethics of the International Society of Ethnobiology [22], and followed all research procedures and protocols at Naturalis Biodiversity Center and Leiden and Wageningen Universities. In Bénin, we obtained a formal invitation from the Faculté des Sciences Agronomiques, Université d’Abomey-Calavi, received formal approval and a research permit (# 041511) from the Faculté des Sciences et Techniques, Université d’Abomey-Calavi, and a plant export permit (#0000591) from the Service de la Protection des Vegetaux et du Control Phytosanitaire, Ministre de l’Agriculture, de l’Elevage et de la Peche. In Gabon, we received a letter of invitation (#176), formal approval, and research permit (#AR0028/12) from the Centre National de la Recherche Scientifique et Technologique (CENAREST), authorization to enter the National Parks (#000026) from the Agence Nationale des Parcs Nationaux (ANPN), and authorization (#00145, #00219) from the Institut de Phamacopee et de Medicine Traditionelles (IPHAMETRA) to export our botanical specimens. Given the ethnobotanical nature of our research, further ethical approval by a bioethics board was deemed not required by these institutions. All data were handled and stored anonymously.

### Study area and sampling

Bénin, with a population of over 9.8 million people, is located in West Africa, between Nigeria and Togo [23]. The main ethnic groups are Fon (39%), Adja (15%), and Yoruba (12%). According to the United Nations Development Program (UNDP), which bases its Human Development Index (HDI) on life expectancy, education, and income, Bénin is considered a country of “low human development” [24]. Its vegetation cover is mainly savanna [25]. Gabon, located in Central Africa, borders the Atlantic Ocean at the Equator, between Republic of the Congo and Equatorial Guinea, and has a population of over 1.6 million people, mainly of Fang, Bapounou, Nzebi, and Obamba ethnic groupings [26]. Gabon is considered by the UNDP to be a country of “medium human development” [27]. It is estimated that up to 80% of Gabon is covered with forest [28]. Both countries, although highly varied in population, level of human development, and vegetation cover, have populations that use traditional medicine as their primary form of healthcare.

The Bénin fieldwork took place between April and October 2011 in the six departments of Kouffo, Zou, Plateau, Ouémè, Atlantique, and Mono. We worked with the major ethnic groups represented in the country, mainly Fon and Yoruba people and related ethnicities. Research in Gabon began in June 2012 and concluded in December 2012, spanning the six departments of Estuaire, Woleu-Ntem, Haut-Ogooué, Ngounié, Moyen-Ogooué, and Ogooué-Ivindo. In Gabon, we worked with Bantu-speaking ethnic groups, namely the Fang, Mitsogo, Obamba, and Bapounou peoples. In each country, we started the data collection at the market, working with willing and knowledgeable herbal medicine saleswomen and then utilized snow-ball sampling to identify additional women in urban and rural communities.

### Ethnobotanical questionnaires

By spending time at the markets and conversing informally with female merchants, we were able to identify local health concerns, commonly utilized species, and respected and knowledgeable collaborators. These activities enabled an emic approach to plants and healthcare and built the trust and mutual understanding necessary to collect data on sensitive information such as sexuality and fertility [29]. This information was used to develop an ethnobotanical women’s reproductive health questionnaire, based on Alexiades’ [30] recommended guidelines for collection of ethnobotanical information. The questionnaires were designed in English and then translated into Beninese and Gabonese French during each fieldwork phase. They consisted of (1) health issue free-listing exercises and (2) open-ended questions inquiring about herbal remedies (plant, use, preparation, and administration) used for statistical causes of maternal mortality and locally-determined health concerns. We conducted a total of 87 questionnaires, 46 in Bénin and 41 in Gabon. The Beninese informants were divided between 42 women and four men, and distributed between 23 market, 17 rural and six urban settings. The Gabonese participants were divided between 40 women and one man, and distributed between 30 rural, six market, and five urban settings. Men were included in the research as informants due to their recognition in their communities as having substantial knowledgeable on the use of plants in women’s reproductive health issues. Participants received monetary compensation for their involvement in the research. Interpreters were employed in situations where participants did not speak French. After introducing ourselves and our research institute, closely explaining the nature of our research, and receiving verbal consent, we conducted the questionnaires in the participants’ own surroundings.

### Plant collection

Directly following each questionnaire, we accompanied informants into the surrounding areas to collect plant species mentioned in the interviews. For questionnaires completed with market sellers, we purchased the cited plant species directly from the market stalls. We used standard ethnobotanical collection methods [30] to allow for an adequate taxonomic identification of the species, and the documentation of local names, recipes, and perceived effects. We collected over 800 plant vouchers and information on their medicinal uses (see Additional file 1 and Additional file 2). Vouchers of all collected plants were deposited at the main herbaria in each country (BEN in Bénin and LBV in Gabon), with a complete set of duplicates stored at the National Herbarium of the Netherlands (WAG), now merged with Naturalis Biodiversity Center.

### Biomedical healthcare provider interviews

We interviewed a total of 18 (six in Bénin and 12 in Gabon) biomedical healthcare providers, including nurses, midwives, doctors, and gynecologists. The interviews took place at national hospitals in urban areas (Cotonou in Bénin and Libreville in Gabon) as well as government and private health clinics in rural communities. These semi-structured interviews included (1) free-listing of salient reproductive health problems, (2) questions related to culturally-bound disease concepts, (3) open-ended questions about practitioners’ experiences with patients who utilized plant-based medicine prior to seeking biomedical care and (4) opinions on the benefits and risks of traditional medicine.

### Data analysis

The ethnobotanical questionnaires were analyzed with three main indices. The first index was the number of times an illness was mentioned in the free-listing exercise. Each informant was asked to give her opinion on the top three health issues that caused the most suffering for women. Secondly, we calculated the knowledge frequency of the informants by averaging the number of citations for each health issue and the percentage of informants who knew at least one herbal remedy for each health condition. Lastly, we calculated the number of plant species cited per health issue, which captured informants’ practices of treating diseases. The health issues with the most cited species were considered to be of high importance to the community, based on the principle that the greater importance of a health condition, the most plant species are used to treat it [31-34]. We summarized the responses of the local biomedical healthcare providers and selected key examples to illustrate their experiences with women who self-treated with medicinal plants prior to arriving at clinics and hospitals.

## Results

### Free-listing analysis

Malaria, pregnancy-related concerns, and infections were the most commonly mentioned health complaints by women in the Beninese free-listing activity (Table 1). Pregnancy-related conditions included a range of concerns such as avoiding miscarriage, managing early pregnancy sicknesses (stomachache, vomiting, and diarrhea), strengthening the fetus, and preparing for childbirth. The statistical causes of maternal health were not strongly reflected in the free-listing activity, with the exception of infections, which may not be directly correlated with the biomedical definition of sepsis. Post-partum hemorrhage ranked sixth among Beninese informants’ concerns, tied with headache. Hypertension was mentioned by only two of the 46 informants.

**Table 1:** Frequency of women’s health complaints cited by 46 informants in Beninese free-listing activity

**Health issue**	**Frequency**
Malaria	0.46
Pregnancy-related	0.33
Infections	0.24
Fever	0.20
Infertility	0.20
Menstrual-related	0.20

Menstrual-related concerns, stomachache, and infertility were the health complaints most frequently cited in the Gabonese free-listing activity (Table 2). Menstrual-related concerns included painful menstruation, black-colored menses, and heavy cramps. Like the informants in Bénin, women in Gabon did not perceive post-partum hemorrhage or high blood pressure as top concerns. Infections were mentioned by two of the 41 informants.

**Table 2:** Frequency of women’s health complaints cited by 41 informants in Gabonese free-listing activity

**Health issue**	**Frequency**
Menstrual-related	0.44
Stomachache	0.41
Infertility	0.22
Packache	0.17
Malaria	0.10
Childbirth-related	0.10
Worms	0.10

### Informants’ knowledge of herbal remedies

Beninese women were most knowledgeable on herbal remedies for pregnancy-related concerns, anemia, high blood pressure, and breast milk stimulation (Table 3). Herbal treatments were administered in pregnancy: (1) to strengthen and protect the fetus (26%), (2) to be consumed as nutritious (plant-based) foods (17%), (3) to prepare the body for delivery (15%), (4) to promote general health and well-being of the mother (13%), (5) to treat/prevent early first trimester illnesses (12%), (6) to treat malaria (6%), and (7) other (fatigue, stomachache, antibiotic, etc.) (11%). Herbal remedies for childbirth-related concerns were mainly reported to be used to facilitate childbirth, but also to assist in the removal of the placenta and for use as a post-birth womb cleanse. The majority of Gabonese women knew herbal remedies for breast milk stimulation, anemia, vaginal cleansing, and menstrual-related concerns (Table 3). Herbal remedies for facilitating childbirth were reported to be used beginning in the seventh month of pregnancy. Of the 41% of informants who knew a treatment for postpartum hemorrhage, half of these responses were for hot water massage, in which herbs were not involved.

**Table 3:** Informant knowledge on women’s health issues in Bénin (46 questionnaires) and Gabon (41 questionnaires)

**Health issue**	**# of citations**	**# of citations**
	**(% informants**^**1**^**)**	**(% informants**^**1**^**)**
	**Bénin**	**Gabon**
Anemia	62 (98%)	63 (88%)
Breast milk stimulation	44 (85%)	78 (93%)
Pregnancy-related	103 (98%)	60 (66%)
Menstrual-related	65 (83%)	53 (76%)
High blood pressure	49 (87%)	43 (63%)
Childbirth-related	70 (78%)	56 (66%)
Vaginal cleanse	39 (67%)	64 (76%)
Sexually transmitted infections	66 (83%)	13 (34%)
Infertility	31 (67%)	39 (46%)
Postpartum hemorrhage	37 (73%)	22 (41%)

¹Percentage of informants with knowledge of at least one treatment.

### Health conditions with the most species

Beninese informants mentioned a total of 248 species for women’s reproductive health (see Additional file 1). More species were cited for pregnancy and menstruation, 36% and 32% respectively, than for other health conditions, followed by anemia (25%) and infertility (23%) (Table 4). Informants mentioned species to treat menstrual-related concerns that concerned length (too long, delayed, irregular), pain (too heavy, too painful), texture (slimly, sticky), color (black, clear) and smell (too odorous). *Sarcocephalus latifolius* (Sm.) E. A. Bruce, was frequently cited as an herbal tea remedy to treat menstrual complications (see Additional file 1).

**Table 4:** Number of species used per health condition in Bénin (46 questionnaires) and Gabon (41 questionnaires)

**Health condition**	**# of species**	**# of species**
	**(% of 248 species)**	**(% of 189 species)**
	**Bénin**	**Gabon**
Pregnancy-related	90 (36%)	41(22%)
Menstrual-related	79 (32%)	28 (15%)
Anemia	62 (25%)	21 (11%)
High blood pressure	39 (16%)	34 (18%)
Infertility	58 (23%)	13 (7%)
Vaginal cleanse	28 (11%)	37 (20%)
Childbirth-related	38 (15%)	25 (13%)
Breast milk stimulation	23 (9%)	29 (15%)
Sexually transmitted infections	40 (16%)	13 (7%)
Postpartum cleanse	28 (11%)	23 (12%)
Postpartum hemorrhage	28 (11%)	12 (6%)

Gabonese informants mentioned a total of 189 species for women’s health (see Additional file 2). Women used 22% of the herbal pharmacopeia for pregnancy, 20% for vaginal cleansing and 18% of species for high blood pressure (Table 4). Breast milk stimulation and menstruation followed, each with 15% of the total numbers of species. Gabonese participants commonly cited the use of the leaves of *Alchornea cordifolia* (Schumach. & Thonn.) Müll. Arg. in direct vaginal insertion for a vaginal cleanse (see Additional file 2). Further analysis on the frequency of species mentioned in our study will be published elsewhere.

### Perspectives of the local biomedical healthcare providers

The Beninese biomedical healthcare providers cited malaria most often as a health threat for pregnant women in the free-listing activity. The Gabonese healthcare providers cited sexually transmitted infections most frequently, followed by stomachache, malaria and infertility. They suggested a strong causal link between infertility and the high number of sexually transmitted infections and clandestine abortions. Biomedical staff in both countries recognized the role of traditional medicine in their patient’s reproductive lives, and shared examples of both positive and negative effects. Doctors in Gabon praised the use of a post-partum hot water massage for mothers’ recovery after childbirth. Staff in private clinics in Bénin mentioned that traditional healers were occasionally called into the clinic to assist in complicated births. However, severe negative effects were also reported, such as the combined use of traditional and modern medicine leading up to childbirth in Bénin. Doctors in Gabon described situations with patients who used plants to speed up contractions that eventually led to uterine rupture.

Although we did not find a strong pattern that biomedical healthcare providers viewed plant-based traditional medicines either negatively or positively for women’s health, both sets of informants clearly conveyed that national policies did not authorize the use of traditional medicine in hospitals. These policies limited the amount of information they were able to share with their patients. They suggested that these restrictions influenced patients’ willingness to discuss their plant use practices with them. Gabonese healthcare providers frequently expressed a concern for the lack of scientific documentation on the effects of medicinal plants and the lack of standard dosage in traditional medicine.

## Discussion

### Locally-perceived health issues

Malaria in pregnancy was commonly cited by women as a health concern in the free-listing activities as well as by the biomedical healthcare providers. International efforts to combat malaria are evidenced in the promotion of malaria prevention therapies for pregnant women by the WHO and the sixth goal of the MDGs [35,36]. Biomedicine recognizes malaria as a serious health threat during pregnancy due to the increased risk of low birth weight and maternal and infant anemia [37-40]. Although malaria is seen as a common concern by local women, local healthcare providers, and (inter) national health organizations, there is little attention from international organizations on the use of plants to treat malaria, especially for pregnant women. Informants in our study were careful to distinguish between plants used for general cases of malaria and those used for pregnant women with this disease. Recent pharmacological research has highlighted the role of medicinal plants in treating malaria in both countries [41,42], but more research is needed to understand the effects of medicinal plant use during pregnancy. We did not systematically ask about malaria in our questionnaires since we did not consider malaria to be a reproductive health issue at the time of designing our questionnaire. This oversight is likely reflected in the low number of plant species cited by informants and is also apparent in international gynecological health programs, as malaria is often not associated with reproductive health.

Menstrual complaints ranked high on the free-listing exercises of both sets of informants. The majority of women knew how to treat menstrual-related conditions and numerous plant species were cited as treatments. Menstruation itself was not considered an illness, but irregularities, pain, and variations in color and smell of blood were frequent concerns. Although not a priority in national or international women’s health agendas, menstrual management has been identified as a priority issue by women across developing regions of the world [21,43,44]. It has direct implications for not only hygiene and infections, but also for productivity and participation in society [45]. Menstruation limits women’s participation in traditional social functions in both Bénin and Gabon. Likewise, as has been documented in Tanzania, menstruation negatively impacts young women’s ability to attend school, resulting in lower attendance and achievement [46]. In Gabon, painful menstruation was linked in cultural terms to infertility. Beninese informants mentioned the correlation between heavy menstruation and anemia as a reason to regulate menstruation. Given the high value placed on fertility in most African societies [47], regular menstruation serves as an indication that a woman can get pregnant; effective menstrual management secures future childbearing [48].

Infertility was among the four most frequently mentioned reproductive health concerns by all women, and ranked high on the tables of participants’ knowledge and plant species. Infertility, especially secondary infertility, has been documented as a reproductive health issue in Sub-Saharan Africa [49,50], as well as a psychological and social concern [51,52]. It has been estimated that up to 30% of couples from sub-Saharan Africa have primary or secondary infertility [53]; a 1983 study showed that 32% of Gabonese women remain childless and the end of the childbearing years, the highest of all African countries involved in the study [54]. Biomedical health providers in Gabon echoed informants’ concerns of infertility and linked it to the high number of sexually transmitted infections and clandestine abortions. Their experiences were supported by a study in eastern Gabon, which found high levels of upper genital tract infections in infertile women [55]. The prevalence of infertility has also been described in recent publications on the use of modern fertility treatments in developing country contexts [56,57]. The WHO published a bulletin in 2010 on the issues facing women in the “infertility belt” of Africa, and called for more available and affordable fertility treatments [58]. Given expenses involved in modern fertility treatments and the social stigma against being infertile, plants offer women an affordable and private way of addressing this ailment [59].

Infertility was also apparent in cultural-bound diseases mentioned by women in both countries. In Bénin, a culturally-bound disease known a “loudjo” (Fon) was cited as a common case for infertility in which a women’s body rejects sperm. Gabonese participants described a cultural bound disease known as “ona” or ‘’onyaboom” (Fang) caused by worms that rest in the womb and cause sterility. Three additional cultural bound diseases were mentioned in the Gabonese fieldwork (see Additional file 2), although they were not the focus of this manuscript, since they were only mentioned by one informant each. These cultural illnesses included “les urines” (Fang/French)- an infection characterized by frequent urination, “zchaw” (Fang)- a gynecological abnormality similar in description to fibroids and cysts, and “mfoes” (Fang)- an illness characterized by back pain. These diseases highlight local understanding of health and are important for biomedical healthcare providers to be aware of in order to have a comprehensive understanding of local healthcare [60].

Pregnancy-related symptoms were common health concerns in both countries. It can be expected that pregnancy has many herbal treatments, due to the numerous stages over nine months in which a woman would seek healthcare. Maintaining a pregnancy and ensuring a safe birth are closely linked to the high social value of fertility [47]. The role of medicinal plants in pregnancy and childbirth has been reflected in other African countries and worldwide [61-64].

Breast-milk stimulation was a common concern for Gabonese informants. The cultural importance of breast milk in Africa is well documented [65-67]. While the WHO does not mention breast milk-related problems in their programming, concerns of inadequate breast milk quantity are a common concern for women worldwide and cited as a reason for not fulfilling the WHO guidelines of six months of exclusive breastfeeding [68]. Shared breastfeeding has been reported as common practice among women in Gabon, with estimates of up to 40% of women engaging in the practice [69].

### Statistical top causes of maternal mortality

Informants in our study did not report the top statistical causes of maternal morbidity and mortality as their most urgent gynecological health concerns. However, over three-fourths of the Beninese informants knew an herbal remedy for treating both high blood pressure and post-partum hemorrhage. Nearly half of the Gabonese informants knew at least one treatment for post-partum hemorrhage and 63% of the women knew plants used to treat high blood pressure. Although further research is needed in order to make more substantial claims, the relative low number of plants cites in our study for postpartum hemorrhage, in particular Gabon, may be reflected in the high rates of maternal mortality associated with hemorrhage. This case reflects a situation where biomedical solutions may be urgently needed in order to improve maternal health in Africa [70]. Some informants were not familiar with hypertension, which is an area where health programs efforts also should improve their educational efforts. It was also not clear whether informants’ concepts of high blood pressure matched biomedical definitions of the illness.

Sepsis, the third most common cause of maternal morbidity and mortality, is a health issue that has not been clearly-defined in health programs worldwide. Many reports suggest that the burden of sepsis on morbidity and mortality are largely underreported [71-73]. Infections, both general and sexually-transmitted, were mentioned by both sets of informants, but sepsis as a distinct category was not cited. It is possible that sepsis is reflected in the numerous recipes and plants used for vaginal cleansing and uterus cleanses. In Gabon especially, vaginal cleanses were a common health practice, with 76% of informants knowledgeable on herbal treatments. Although these cleanses may not be directly associated with sepsis or infections, they may have a role in either preventing vaginal infections or increasing infections by disturbing vaginal flora. Although some research has explored the role of intravaginal practices in increased HIV infections [15-17,74], more research is needed to draw further conclusions on the role of vaginal cleansing in women’s reproductive healthcare, and more generally sepsis and infections.

The statistical causes of maternal mortality were also not reported by the local biomedical staff as urgent gynecological health concerns. This outcome could be explained by the generalized nature of African maternal mortality statistics [3,4], which may not accurately reflect the health status of the populations with which we worked. The reliability of these numbers as an accurate measure of a population’s health also comes into question, as recent literature on African economic statistics has proposed [75]. The differences in perspectives nevertheless highlight key conceptual differences on well-being, health, and illness. Awareness of these differences can improve healthcare for African women by enhancing educational efforts and designing health initiatives that are culturally-appropriate to local communities.

### Key insight from local healthcare providers

The local doctors, midwives, and gynecologists embody the biomedical perspective on the local level. They work at the interface between the local women’s practices and perceptions and the biomedical science promoted in government hospitals and clinics. The biomedical staff in our study recognized the role of plant-based traditional medicine in their patients’ lives, including the benefits and the risks, but were unable to make recommendations or offer medical advice due to the lack of documentation on plants’ effects and the restrictions of national health policies. Local biomedical staff are uniquely positioned at the intersection of the two medical systems [76], a role that deserves further research and analysis in order to understand how the pluralistic medical system can better serve women in Africa.

### Strength and weakness of our research

Our methodology of using three indices to capture the perceptions, knowledge, and plant use patterns enabled a triangulation of the most salient health concerns for the informants involved in this study. While we are confident that our results reflect the views of those individuals involved in our research, both countries have diverse ethnicities whose knowledge and perceptions were not represented in this study. Additional studies are needed to avoid generalizing our results to the entire female Beninese and Gabonese populations and to address differences in health perceptions between ethnic groups. Likewise, our use of snowball sampling resulted in uneven numbers of women from urban, rural, and market settings and the knowledge of several men. Our aim was not to make comparisons between marketplaces and rural and urban settings, but to capture local women’s perceptions and routine practices. Five men were included in the study since they were recognized in their communities as being knowledgable on plants used for women’s reproductive health. Although we did not have a large enough sample to make a statistical comparison, we found that men often had specialist knowledge on issues such as female infertility and complicated births. Future research can investigate differences in knowledge between the sexes and among varied settings.

## Conclusion

In the diverse settings of Bénin and Gabon, plants serve as an entry point to understanding salient gynecological concerns and common health practices. Plants and women’s knowledge contribute to the local population’s management of the top statistical causes of maternal mortality, but other health conditions such as menstruation and infertility were more salient health concerns. Echoing the stances of the biomedical staff of both countries, more research is needed on the role of plants in women’s gynecological healthcare. A renewed commitment to strengthening national policies on traditional medicine could improve the health services offered to women, helping to avoid the adverse effects of combing both systems, and further realize the goal of African women and (inter) national health programs alike of reducing maternal morbidity and mortality.

## Abbreviations

MDGs: Millennium development goals; BEN: National Herbarium of Bénin; LBV: National Herbarium of Gabon; WAG: Former Wageningen University branch of the National Herbarium of the Netherlands, now merged with Naturalis Biodiversity Center; WHO: World Health Organization.

## Competing interests

The authors declare that they have no competing interests.

## Authors’ contributions

AMT carried out the ethnobotanical questionnaires, conducted the biomedical health care provider interviews, collected and identified the plants, analyzed the data, and drafted the manuscript. TvA conceived of the study, acquired funding, participated in its design and coordination, helped to identify the plants, and helped to draft the manuscript. Both authors read and approved the final manuscript.

## Authors’ information

AMT is a PhD student at Leiden University, Naturalis Biodiversity Center in the Netherlands. TvA is a postdoc researcher at Leiden University, Naturalis Biodiversty Center in the Netherlands.

## Pre-publication history

The pre-publication history for this paper can be accessed here:

http://www.biomedcentral.com/1472-6882/14/113/prepub

## Supplementary Material

Additional file 1 **Species cited in 46 questionnaires in Bénin.** Scientific botanical name, name in local language(s), used plant part, preparation, use category and AMT collection number. Click here for file

Additional file 2 **Species cited in 41 questionnaires in Gabon.** Scientific botanical name, name local language(s), used plant part, preparation, use category and collection number. Click here for file
