# Supplementary material for: Comparing local perspectives on women’s health with statistics on maternal mortality: an ethnobotanical study in Bénin and Gabon
Source: BMC Complement Altern Med. 2014 Mar 28;14:113. doi: 10.1186/1472-6882-14-113 (PMC3986651; doi:10.1186/1472-6882-14-113)
Supplement: Additional file 1 — Species cited in 46 questionnaires in Bénin. Scientific botanical name, name in local language(s), used plant part, preparation, use category and AMT collection number. [file 1472-6882-14-113-S1.pdf]

Additional file 1

Species cited in 46 questionnaires in Bénin: scientific botanical name, name in local language(s), used plant part, preparation, use category and AMT collection number

Botanical Name	Local Name[a]	Used part	Preparation[b]	Use category[c]	AMT#[d]
Abrus precatorius L.	degbeybegbey (F), yekpeyekpeman (F), viviman (N)	root, leaves	T	contraception, infertility, pregnancy	NC
Acacia nilotica (L.) Delile	bonni (F)	seeds	T	menstruation	338
Acanthospermum hispidum DC.	togbama (M), kpononmi (N)	plant, leaves	T	HBP, infertility, pregnancy	376, 381, 535
Acmella caulirhiza Delile	awlekpekpe (S)	plant	T	postpartum hemorrhage	397
Acridocarpus smeathmannii (DC.) Guill. & Perr.	gbanguinan (F)	root, bark	T	anemia	300, 355, 599
Acrostichum aureum L.	Sofoco (S)	leaves	T	pregnancy	NC
Aframomum melegueta K.Schum.	atakoun (F)	fruit	T	childbirth, galactagogue, menstruation, STIs	309
Afzelia africana Pers.	aguakpogoto (F)	bark	VW	STIs	362
Aganope stuhlmannii (Taub.) Adema	siensiendo (F)	root, bark	T	infertility, menstruation	NC
Agelaea pentagyna (Lam.) Baill.	ahouanhlazou (S)	leaves	T	pregnancy	383
Albizia adianthifolia (Schum.) W.Wight	awagotingoto (F)	bark	T	menstruation	369
Allium cepa L.	petite onion (Fr)	stem	V, T, VW, A	galactagogue, HBP, infertility, menstruation, postpartum infections	117
Allium sativum L.	aiyo (G)	stem	T, D	childbirth, cysts, fibroids, HBP, menstruation	NC
Aloe macrocarpa Tod.	aloes (M)	leaves	J	anemia	487
Ampelocissus leonensis (Hook.f.) Planch.	tekple (N)	plant	T	infertility, pregnancy	320
AMT 271	indi (T)	root	T	postpartum infections	271
Anacardium occidentale L.	cadjou (F)	bark, leaves, root	T, VW, HB	HBP, infertility, STIs, postpartum infections, pregnancy	331
Ananas comosus (L.) Merr.	ananas (Fr)	fruit	T	anemia	NC
Anchomanes cf. *difformis* (Blume) Engl.	agohouéhè do (F)	root	T	infertility	315
Annickia polycarpa (DC.) Setten & Maas	atahé (F)	bark	T	menstruation	NC

Botanical Name	Local Name[a]	Used part	Preparation[b]	Use category[c]	AMT#[d]
Annona muricata L.	shapshap (F)	leaves	T	HBP, STIs	NC
Annona senegalensis Pers.	nyuglo (F), bejunongley (M), tineybobo (T)	leaves, root	E, P, T	childbirth, STIs, pregnancy	602, 644
Anthocleista sp.	clabalabagoto (F)	bark	T	intestinal cleanse	523
Anthocleista vogelii Planch.	gotoundo (F)	root, leaves, wood	T	infertility, intestinal cleanse, stomachache	348
Arachis hypogaea L.	arachide (Fr)	seeds	E	galactagogue, pregnancy	NC
Argemone mexicana L.	wetcheyon (G)	leaves	T, VW	pregnancy, STIs, vaginal cleanse	NC
Artocarpus cf. *altilis* (Parkinson ex F.A.Zorn) Fosberg	blèfutu asu (M)	leaves	T	HBP	NC
Asteraceae sp.	atentebe (T)	plant	T	stomach ache	292
Azadirachta indica A.Juss.	kini (F)	leaves	T	abortion, pregnancy, stomachache	NC
Baphia nitida Lodd.	sokpèkpè (F)	wood	T, A	contraception, cysts, fibroids, intestinal cleanse, menstruation, postpartum cleanse	319
Barteria cf. *nigritana* Hook.f.	oko goto (F)	bark		anemia	NC
Bauhinia thonningii Schum.	kloman (F), banmo (T)	leaves	E, T	pregnancy, HBP	647
Beta vulgaris L.	la betterave (Fr)	root	E	anemia	NC
Blighia cf. *unijugata* Baker	agboviawondo (N)	bark	T	intestinal cleanse	NC
Boerhavia erecta L.	tikpatikpalala (T)	leaves	E	pregnancy	298
Bridelia ferruginea Benth.	houssoukokoé (F)	root, leaves, bark	T, A, VW	anemia, contraception, infertility, menstruation, postpartum infections, STIs, vaginal cleanse	NC
Bryophyllum pinnatum (Lam.) Oken	afitiman (S)	leaves	T	postpartum cleanse	NC
Burkea africana Hook.	atapa (T)	leaves	E	pregnancy	646
Caesalpinia bonduc (L.) Roxb.	adjikuiman (F)	seeds, leaves, root	D, E,T, W	childbirth, diuretic, infertility, menstruation, postpartum bleeding, postpartum cleanse, postpartum infections, pregnancy, STIs, vaginal cleanse	484, 651
Caesalpinia pulcherrima (L.) Sw.	orgueil de chine (Fr)	fruit (young), leaves, bark	T, P	HBP, infections	480
Cajanus cajan (L.) Millsp.	eweotini (N)	leaves	T	menstruation	NC
Caladium bicolor (Aiton) Vent.	wèkènoukoun (F)	plant	TC	menopause cause	568
Calotropis gigantea (L.) Dryand.	kpinto (F)	leaves	T	cough	NC
Canna cf. *indica* L.	sidakin (G)	plant	T	STIs	NC
Capsicum annuum L.	pimment (Fr)	fruit	T, P, EN, A	cysts, fibroids, hemorrhoids- internal, HBP, intestinal cleanse	256
Carica papaya L.	kpinman (F,G)	leaves, fruit, root	T, D	anemia, galactagogue, infertility, contraception, cysts, fibroids, HBP, pregnancy, menstruation, vaginal cleanse	NC
Carissa spinarum L.	ahouanzodo (F)	root	T, VW, A	anemia, cysts, fibroids, menstruation, postpartum	332,

Botanical Name	Local Name[a]	Used part	Preparation[b]	Use category[c]	AMT#[d]
				infections, STIs	373, 504, 524
Cassytha filiformis L.	agbégbékan (F)	plant	T, VW	pregnancy, vaginal cleanse, infertility	326
Ceiba pentandra (L.) Gaertn.	kpatindèhoun (F), batidenia (M)	leaves	D	childbirth	567
Celosia cf. *argentea* L.	soman (G)	leaves	T	anemia	NC
Celtis cf. *zenkeri* Engl.	agbohingla (S)	bark	T	cysts, fibroids	NC
Ceratotheca sesamoides Endl.	komokuile (T), agboma (F)	leaves, whole plant	D, E, T	anemia, childbirth, pregnancy	655
Chamaecrista mimosoides (L.) Greene	kilafimitche (S)	plant	VW	STIs	389
Chamaecrista rotundifolia (Pers.) Greene	azima (F, G)	leaves, whole plant	VW, T	infertility, pregnancy, STIs	NC
Chassalia kolly (Schumach.) Hepper	djêtindo (F)	root	T	childbirth, intestinal cleanse, cysts, fibroids, menstruation	187, 328
Chromolaena odorata (L.) R.M.King & H.Rob.	agatou (F, G, N)	leaves	T, VW	vaginal cleanse	NC
Chrysophyllum albidum G.Don	azongogoegoto (G)	bark	T	galactagogue	NC
Chrysopogon sp.	tékanwannon (F)	plant	T	menstruation	339
Cissampelos mucronata A.Rich	djokodje (K)	leaves	T	postpartum hemorrhage	515
Cissampelos mucronata A.Rich.	djokodjè (F, G)	leaves, whole plant	T	infertility, CBD loudjo, infertility, menstruation, pregnancy	314
Cissampelos owariensis P.Beauv. ex DC.	tjokodje (F)	leaves	D	infertility, pregnancy	NC
Cissus populnea Guill. & Perr.	dedo (F)	root	D	childbirth	122
Citrullus colocynthis (L.) Schrad.	tchègba (F), kaka (T)	leaves, seeds, fruit	E, T	STIs, intestinal cleanse, pregnancy	238, 303
Citrullus lanatus (Thunb.) Matsum. & Nakai	wanyiwanyikoun (F)	fruit	T	intestinal cleanse	505
Citrus aurantiifolia (Christm.) Swingle	clé (F, G)	leaves, fruit, root	E, EA, T	childbirth, galactagogue, HBP, intestinal cleanse, menstruation, pregnancy, breast inflammation, postpartum cleanse, postpartum infections, infertility	345
Citrus sp.	klèman (F)	leaves, root, fruit	T	HBP, menstruation, pregnancy, intestinal cleanse, menstruation, stomachache	347
Clausena anisata (Willd.) Hook.f. ex Benth.	gbozohoun (F)	leaves, bark, root	VW, T	menstruation, postpartum infections, STIs	330, 576
Cleistopholis patens (Benth.) Engl. & Diels	hounsoué do (F, G, N)	bark, leaves, root	T	infertility, intestinal cleanse, pregnancy	375

Botanical Name	Local Name[a]	Used part	Preparation[b]	Use category[c]	AMT#[d]
Cleome viscosa L.	agatouma (F)	plant	VW	STIs	NC
Cnestis ferruginea Vahl ex DC.	akpaflo (F)	seeds, root	T	menstruation, anemia	340
Cochlospermum planchonii Hook.f. ex Planch.	betou (T)	leaves	E	pregnancy	656
Cocos nucifera L.	agodo (F, G, N)	fruit, root, seeds, fruit water	D, T	anemia, galactagogue, cysts, fibroids, HBP, yellow fever, menstruation	370, 378
Combretum cf. grandiflorum G.Don	adounsitoman (G, S)	leaves	D, T	childbirth, infertility	NC
Commelina erecta L.	tchankoko (F)	rhizome	T	menstruation	334
Corchorus olitorius L.	krenkren (F)	leaves, root	E, T	food, menstruation	166
Costus lucanusianus J.Braun & K.Schum.	tetregoudou (G)	plant	T	cysts, fibroids	372
Costus sp.	tetregouman (S)	leaves	T	intestinal cleanse, vaginal cleanse	NC
Crateva adansonii DC.	hontonzonzouin (F)	leaves	T, VW	HBP, menstruation, STIs, vaginal cleanse	NC
Crescentia cujete L.	kamma (F)	leaves	D	intestinal cleanse	NC
Croton gratissimus Burch.	jelele (F)	leaves, bark, root	T, P	CBD loudjo, childbirth, HBP, menstruation, pregnancy	327
Cucumeropsis cf. mannii Naudin	goussitchègba (F, G)	seeds	T	infertility, intestinal cleanse, menstruation	NC
Curculigo pilosa (Schumach. & Thonn.) Engl.	ayote (F)	tuber	V, D, T	galactagogue, intestinal cleanse, menstruation	118, 333
Curcuma sp.	chyiaoumkoko (F)	rhizome		infections	196
Cyanthillium cinereum (L.) H.Rob.	hunsikusey (F)	plant, leaves	T	CBD loudjo, contraception, pregnancy,	540, 595
Cymbopogon citratus (DC.) Stapf	teeman (F, N)	leaves	T	anemia, childbirth, galactagogue, pregnancy, stomachache	NC
Cynometra megalophylla Harms	bougoto (F, N, G)	bark	T	anemia	NC
Daniellia oliveri (Rolfe) Hutch. & Dalziel	nyado (F), inya (T)	leaves, bark, wood	E, D, HB, P	pregnancy, galactagogue, infertility	584, 652
Desmodium gangeticum (L.) DC.	zèdali (F, N, G)	leaves, whole plant	T, D	childbirth, infertility, pregnancy	316
Desmodium velutinum (Willd.) DC.	bandowo (F, N, G)	leaves, whole plant, fruit	T, VW	CBD loudjo, HBP, menstruation, pregnancy	250, 508
Dialium guineense Willd.	loma (M)	leaves	D, E	galactagogue, intestinal cleanse, vegetable	481
Dichapetalum madagascariense Poir.	gbago (F)	leaves	T	cysts, fibroids, HBP, pregnancy, menstruation	142, 169
Diodella sarmentosa (Sw.) Bacigalupo & Cabral ex Borhidi	sèhwi (F, G)	plant, leaves	T	infertility, menstruation, pregnancy	317, 528
Dioscorea sp.	gando (F)	root, fruit	T	cysts, fibroids, menstruation, anemia	357

Botanical Name	Local Name[a]	Used part	Preparation[b]	Use category[c]	AMT#[d]
Dysphania ambrosioides (L.) Mosyakin & Clemants	godo (F), amantrouzou (F)	plant, leaves	EA, VW, T	menstruation, postpartum infections, STIs, vaginal cleanse	479, 519
Ehretia cymosa Thonn.	mionman (F), bodomey (T)	leaves	E, T	menstruation, pregnancy, HBP	380, 657
Elaeis guineensis Jacq.	l`huile rouge (Fr)	seeds, oil from seeds, infructescence, leaves	E, EA, HB, T	galactagogue, internal hemorrhoids, HBP, muscle pain, fatigue, pregnancy	578
Eleusine indica (L.) Gaertn.	akpi (K)	leaves	D	childbirth	513
Entada africana Guill. & Perr.	kpakpassoumehaman (S)	leaves	T	contraception	394
Entada gigas (L.) Fawc. & Rendle	gbagbara (F)	seeds, bark	T	infertility, pregnancy	NC
Erythrina senegalensis DC.	pbaklesido (F)	bark, root	T	anemia, contraception, infertility, menstruation pregnancy	131, 527
Fabaceae sp.	fonvi (S)	plant	T	menstruation	NC
Ficus sur Forssk.	agpoto (T)	root, leaves	T	anemia, pregnancy	293, 388, 510, 653
Flacourtia indica (Burm. f.) Merr.	gbohouncadjè (F)	root, leaves	T, D	anemia, pregnancy	127, 356
Flacourtia sp.	gbougbadjo (G)	wood	T	intestinal cleanse	371
Flueggea virosa (Roxb. ex Willd.) Royle	tcheke-tcheke (F)	leaves	T	fever, intestinal cleanse, malaria, pregnancy	193
Fulvifomes cf. *fastuosus* (Lév.) Bondartseva & S. Herrera	chutin (F)	fungus	T	infertility	123
Ganoderma sp.	djouatin (F)	fungus	T	pregnancy	NC
Garcinia kola Heckel	ahowo (G)	wood	T	menstruation	368
Garcinia sp.	kola (F)	bark, seeds	T, E	anemia	NC
Gardenia ternifolia Schumach. & Thonn.	adakpla (F)	plant, root, leaves	T	HBP, malaria, pregnancy	301, 510, 521, 564
Gladiolus dalenii Van Geel	baka (F)	stem	T	menstruation	335
Gmelina arborea Roxb.	fofitin (F)	leaves	T	HBP	359
Gomphrena celosioides Mart.	papatajè (K)	plant	T	HBP	516
Gossypium barbadense L.	tchekey	plant			612
Gossypium hirsutum L.	avokanfochekey (F)	leaves	D, T	anemia	NC
Grewia cf. *carpinifolia* Juss.	oriman (G)	leaves	T	pregnancy	NC
Gymnosporia senegalensis (Lam.) Loes.	yedoman (F)	leaves, bark	T	cysts, fibroids	NC
Helianthus sp.	botiowo (T)	leaves	EN	intestinal cleanse	278

Botanical Name	Local Name[a]	Used part	Preparation[b]	Use category[c]	AMT#[d]
Heliotropium indicum L.	koklusu danpaja (M)	plant, leaves	D, T	childbirth, HBP	522
Heterotis rotundifolia (Sm.) Jacq.-Fél.	hêhêman (F)	leaves, bark	T, D, VW	anemia, childbirth, cysts, fibroids, HBP, infertility, menstruation, pregnancy, protection against sorcery, postpartum infections, STIs	NC
Hibiscus acetosella Welw. ex Hiern	podey (M)	leaves	T	anemia	151
Hibiscus sabdariffa L.	bissap (Fr)	leaves	T	cysts, fibroids, pregnancy	485
Hibiscus surattensis L.	kpofin (F, G, N)	plant, leaves	T	anemia, infertility, pregnancy	150, 310, 503
Hymenocardia acida Tul.	fefeya (T), orukpa (T)	leaves	E	pregnancy	641, 649
Hyptis suaveolens (L.) Poit.	kulubi (T)	leaves, root	E, T	galactagogue, STIs, pregnancy, stomachache	275, 536
Imperata cylindrica (L.) Raeusch.	cekunu (F), eweekan (N)	leaves	VW	pregnancy, vaginal cleanse	NC
Indigofera hirsuta L.	zogbeyzi (M)	leaves	T	pregnancy	170
Indigofera pulchra Willd.	azima (F)	plant	T	pregnancy	614
Ipomoea aquatic Forssk.	ahinandje (S)	plant	T	postpartum hemorrhage	399
Ipomoea batatas (L.) Poir.	patat douce (Fr)	leaves	T	contraception	NC
Irvingia gabonensis (Aubry-Lecomte ex O'Rorke) Baill.	bègbègma (M), asroma (M)	leaves	T	diarrhea, dysentery, hemorrhoids	482
Jatropha curcas L.	yinkpotin (N), eweakporo (N)	leaves	T, HB, VW	anemia, HBP, malaria, menstruation, stomachache	263, 483
Jatropha gossypiifolia L.	yokpotinmannovo (N)	root	T	anemia	353, 572
Justicia flava (Vahl) Vahl	tchoutchougboutchou (F, S)	leaves, whole plant	T	anemia, menstruation	393
Khaya senegalensis (Desv.) A.Juss.	zouzou (F), kasesral (F)	bark, leaves	A, T, D, HB, VW	abortion, anemia, cysts, fibroids, HBP, infertility, menstruation, postpartum cleanse, postpartum infections, pregnancy, STIs, stomachache, vaginal cleanse	121, 284, 526
Kigelia africana (Lam.) Benth.	gnanblikpo (F)	leaves, bark, fruit	EA, T	breast inflammation, cysts, fibroids, infertility, intestinal cleanse, menstruation, stomachache	249, 342, 396
Lannea acida A.Rich.	akou (T)	bark	T	anemia	282
Lannea barteri (Oliv.) Engl.	houmansitékannon (F)	bark, leaves	T	anemia, menstruation	351
Lantana camara L.	hlatchayo (F)	leaves, whole plant	T, VW	postpartum infections, STIs, vaginal cleanse	NC
Laportea aestuans (L.) Chew	kesukesu (F)	plant	T	menstruation	111
Lippia multiflora Moldenke	yinya (F)	leaves	T, VW	cysts, fibroids, STIs, vaginal cleanse	311
Lippia sp.	aglala (F)	leaves	T, VW	HBP, menstruation, postpartum infections, STIs, vaginal cleanse	NC

Botanical Name	Local Name[a]	Used part	Preparation[b]	Use category[c]	AMT#[d]
Lycopodiella cernua (L.) Pic. Serm.	hingble (F, S)	plant	P, T	childbirth, pregnancy	132
Mallotus oppositifolius (Geiseler) Müll.Arg.	adji (T), cecewima (M)	plant, leaves	E, T	intestinal cleanse, postpartum cleanse, postpartum infections	168
Mangifera indica L.	amangagoto (F, G, N)	leaves, bark	HB, T	anemia, pregnancy	191, 382
Manihot esculenta Crantz	kootema (F)	leaves	D	anemia	NC
Melaleuca leucadendra (L.) L.	kpimansin semeton (S)	leaves	D	childbirth	395
Merremia tridentata (L.) Hallier f.	fakale (G)	leaves	T	infertility	NC
Milicia excelsa (Welw.) C.C.Berg	lokoma (F)	leaves	T	contraception	NC
Millettia thonningii (Schum. & Thonn.) Baker	assandjouman (S)	leaves	T	postpartum hemorrhage	398
Millettia thonningii (Schum. & Thonn.) Baker	assoinssoin (F), otietie (G, N)	leaves, bark	T	anemia, galactagogue, infertility, intestinal cleanse, pregnancy, vaginal cleanse	501
Mimosa quadrivalvis var. *leptocarpa* (DC.) Barneby	ahossiboasa (F)	leaves	T, D	HBP, childbirth, HBP, pregnancy	302, 581
Mitragyna inermis (Willd.) Kuntze	lagpatima (M)	leaves	T	STIs	171
Momordica charantia L.	gninsinkin (F)	plant, fruit, leaves	EA, E, T, VW	abortion, anemia, contraception, HBP, intestinal cleanse, postpartum infections, STIs, pregnancy, vaginal cleanse	361
Monodora myristica (Gaertn.) Dunal	sassalikoun (F)	bark, seeds	A, T, V, VW	anemia, galactagogue, infertility, menstruation, postpartum infections, STIs	119, 184, 308
Morinda lucida Benth.	koinsido (F)	leaves, root, bark	A, T	abortion, anemia, contraception, cysts, fibroids, intestinal cleanse, postpartum cleanse, menstruation	112, 321
Moringa oleifera Lam.	kpatiman (F, G), patovide (M)	leaves, bark	E, P, T, VW	childbirth, cysts, fibroids, HBP, STIs, pregnancy	NC
Newbouldia laevis (P.Beauv.) Seem.	dèsèsiigièma (F), akoko (N)	leaves	T, D	anemia, HBP, pregnancy	NC
Nicotiana cf. *tabacum* L.	azoma (F)	leaves	V	galactagogue	NC
Ocimum americanum L.	hissihissi (F)	plant, leaves	T, HB, VW	postpartum infections, pregnancy, STIs, vaginal cleanse	701
Ocimum basilicum L.	kesukesu (M)	leaves	T	menstruation	143
Ocimum gratissimum L.	tchayo (F, G), koumoba (T)	leaves, whole plant	D, T, VW, HB	anemia, breast milk purifier, contraception, intestinal cleanse, menstruation, postpartum cleanse, postpartum infections, pregnancy, STIs, vaginal cleanse	NC
Ocimum sp.		plant, leaves	E, EA, T, D, VW	anemia, fatigue, HBP, infertility, intestinal cleanse, menstruation, muscle pain, postpartum infections, pregnancy, STIs, vaginal cleanse, vaginal infections	NC
Olax subscorpioidea Oliv.	mitindo (F, G, N)	root	T	cysts, fibroids, infertility, intestinal cleanse, menstruation	329

Botanical Name	Local Name[a]	Used part	Preparation[b]	Use category[c]	AMT#[d]
Oldenlandia affinis (Roem. & Schult.) DC.	ahonhoun (F, G)	plant, leaves	T	childbirth, infertility	206, 313
Parinari curatellifolia Planch. ex Benth.	iyafo (T)	leaves	E	pregnancy	650
Parkia biglobosa (Jacq.) G.Don	ahouagoto (F)	leaves, bark, seeds	T, D, P, EN	hemorrhoids-internal, HBP, postpartum cleanse	384
Paullinia pinnata L.	hèdoulifindo (F)	root, leaves	A, E, D, T, VW	anemia, contraception, HBP, menstruation, pregnancy	125
Pavetta corymbosa (DC.) F.N.Williams	lohou (F)	root, leaves	D, T, VW	infertility, pregnancy, vaginal cleanse	312
Pennisetum cf. *glaucum* (L.) R.Br.	mil (Fr)	leaves	D, T	anemia	NC
Peperomia pellucida (L.) Kunth	fifaman (F)	leaves	T	infertility	323
Pergularia daemia (Forssk.) Chiov.	abognfufu (T)	leaves	E	galactagogue, pregnancy	283, 640
Periploca calophylla (Baill.) Roberty	honman (F)	leaves	T	pregnancy	NC
Persea americana Mill.	avocaman (F, G)	leaves	T, D	anemia, HBP, malaria, pregnancy	NC
Phaulopsis ciliata (Willd.) Hepper	chouchougrouchou (F)	plant		protection against spirits	200
Phyllanthus amarus Schumach. & Thonn.	hlinwé (F, G)	plant, leaves	E, T, VW	HBP, intestinal cleanse, menstruation, postpartum cleanse, pregnancy	239, 344, 642
Phyllanthus muellerianus (Kuntze) Exell	agemukogu (T)	root	T	anemia	296
Phymatosorus scolopendria (Burm. f.) Pic. Serm.	degoma (F, G, N)	leaves	VW, T	STIs, pregnancy	120, 377
Physalis cf. *angulata* L.	korogba (N)	plant	T, VW	STIs, vaginal cleanse	NC
Piper guineense Schumach. & Thonn.	piment du guinea (Fr)	fruit, leaves	E, T	contraception, CBD loudjo, pregnancy	NC
Platostoma africanum P.Beauv.	koumobaokuta (T)	leaves	E	pregnancy	648
Polygala arenaria Willd.	mli (F)	plant		infection	205
Portulaca oleracea L.	denkama (F)	plant	EA	pregnancy	110
Prosopis africana (Guill. & Perr.) Taub.	kakema (F)	plant	T, HB, VW	postpartum infections	NC
Psidium guajava L.	guave (Fr)	fruit	E	pregnancy	NC
Pterocarpus erinaceus Poir.	kosso (G, S), eweakpekpe (N)	bark, leaves	T	anemia, cysts, fibroids, menstruation	379
Pterocarpus santalinoides DC.	gbètin (F)	leaves	T	infertility, stomachache	NC
Pycnanthus angolensis	yayado (F)	root	T	anemia	NC

Botanical Name	Local Name[a]	Used part	Preparation[b]	Use category[c]	AMT#[d]
(Welw.) Warb.					
Raphia sp.	dema (F, M)	wood, leaves	T	placenta removal	NC
Rauvolfia vomitoria Afzel.	vonmausin (G)	leaves	T	pregnancy	374
Rhaphiostylis beninensis (Hook.f. ex Planch.) Planch. ex Benth.	kplakplakando (F)	wood, leaves, root	T, D	intestinal cleanse, menstruation, postpartum cleanse	129 500
Ricinus communis L.	tondedji (F)	leaves	D	abortion, intestinal cleanse, menstruation	NC
Rourea coccinea (Schumach. & Thonn.) Benth.	vikplomba (F, G, S)	leaves	T, E	anemia, infertility	167
Sabicea calycina Benth.	aviama (F, S)	plant, leaves	VW	STIs, pregnancy	391
Saccharum officinarum L.	canne asucre (Fr)	stem	T	anemia	NC
Sansevieria sp.	kpoyando (G)	root	T	infertility	NC
Sarcocephalus latifolius (Sm.) E.A.Bruce	kodo (F, G), umbesi (T)	root	T, M, D	abortion, anemia, childbirth, cysts, fibroids, galactagogue, infertility, intestinal cleanse, menstruation, postpartum cleanse, postpartum infections, pregnancy, stomachache	295
Schwenkia americana L.	zron (F)	plant	D, T	childbirth, pregnancy	324, 352
Secamone afzelii (Roem. & Schult.) K.Schum.	zoucoutou (F), anonsiman (F)	plant, leaves	E, T	anemia, galactagogue, infertility, postpartum infections, pregnancy	322, 349, 639, 597
Securidaca longipedunculata Fresen.	abiwèrè (F, G, N)	leaves	T, D, VW	childbirth, infertility, menstruation, postpartum cleanse, pregnancy, vaginal cleanse	318, 336
Senna alata (L.) Roxb.	amasou (F)	leaves, flower	T, A	cysts, fibroids, intestinal cleanse, menstruation	343, 502
Senna italica Mill.	agwègbé (F)	leaves	D, T	constipation, intestinal cleanse	307
Senna obtusifolia (L.) H.S.Irwin & Barneby	kpanwoun (S)	leaves	T	anemia	NC
Senna occidentalis (L.) Link	agonlika (F), ajambulu (T)	leaves	EN	intestinal cleanse	241
Senna siamea (Lam.) H.S.Irwin & Barneby	cassia (F, M)	leaves	T, HB	abortion, childbirth, postpartum cleanse, CBD loudjo, menstruation, pregnancy	165
Sesamum indicum L.	sesame (Fr)	plant, seeds	E, A	menstruation, postpartum infections, pregnancy	NC
Sida acuta Burm.f.	agbegbema (F), etchokotou (G, N)	leaves, root, whole plant	D, E, T, VW	contraception, intestinal cleanse, menstruation, postpartum cleanse, pregnancy	509, 534, 611
Sida cf. *cordifolia* L.	agbidi (F)	leaves	T	postpartum hemorrhage	NC
Smilax anceps Willd.	aglbaliklaklan (F)	root		cysts, fibroids, menstruation	186
Solanum aethiopicum L.	gblèman (F)	leaves	EA	STIs, vaginal cleanse	566
Sorghum arundinaceum (Desv.) Stapf	jehooma (M)	leaves	T	STIs	172

Botanical Name	Local Name[a]	Used part	Preparation[b]	Use category[c]	AMT#[d]
Sorghum bicolor (L.) Moench	adako (F), okaono (T)	leaves	T	anemia, menstruation	247
Sorghum sp.	le sorgho (Fr)	seeds	EA	infections	NC
Spathodea campanulata P.Beauv.	adade (G, N, S)	plant, leaves, bark	T, VW	HBP, infertility, menstruation, pregnancy, STIs	363, 390
Spondias mombin L.	akikon (F)	leaves, bark	T, VW	HBP, malaria, menstruation, vaginal cleanse	128, 360, 494, 635
Stachytarpheta cayennensis (Rich.) Vahl	alotrohe (S)	plant	T	menstruation	387
Stereospermum kunthianum Cham.	adjadey (T)	leaves, root	E, CS	pregnancy	643
Strophanthus hispidus DC.	tchakpa (F), inchao (T)	root, leaves	E, T	childbirth, cysts, fibroids, menstruation, pregnancy, stomachache	277, 350, 358
Struchium sparganophorum (L.) Kuntze	acodigoue (S)	plant	T	anemia	385
Syzygium aromaticum (L.) Merr. & L.M.Perry	atinkingbadota (F)	flower buds	T, V, A, VW	anemia, galactagogue, contraception, infertility, intestinal clenase, menstruation, postpartum infections, STIs	116
Syzygium guineense (Willd.) DC.	mlammido (N)	root	VW	vaginal cleanse	NC
Tapinanthus globiferus Tiegh.	hansimlin (F)	leaves	T	anemia	571
Tectona grandis L.f.	teckdo (F)	leaves, root	HB, T	anemia	NC
Terminalia glaucescens Planch. ex Benth.	alotoun (F)	root, leaves	T, VW	intestinal cleanse, menstruation, vaginal cleanse	305, 627
Tetrapleura tetraptera (Schum. & Thonn.) Taub.	lindja (F)	fruit	VW, T	infertility, menstruation, postpartum infections, STIs, vaginal cleanse	304
Tridax procumbens (L.) L.	kpokpo (F, G)	plant	T	anemia, cysts, menstruation	512
Uvaria chamae P.Beauv.	aylahado (F)	root, leaves	T	anemia, infertility, menstruation, pregnancy	126, 529
Vepris verdoorniana (Exell & Mendonça) Mziray	agbede (S)	leaves	T, VW	anemia, contraception, infertility, menstruation	386
Vernonia amygdalina Delile	amavive (F)	leaves, whole plant	E, HB, T	galactagogue, postpartum infection, pregnancy, vaginal cleanse	491, 645
Vitex doniana Sweet	foman (F)	leaves, root	T, A	hemorrhoids, menstruation	570
Waltheria indica L.	atasu yonunuvima (F), misomituwey (M), kasa (T)	Plant, leaves, root	D, T	anemia, infertility, intestinal cleanse, menstruation, postpartum hemorrhage	130, 253, 565
Xylopia aethiopica (Dunal) A.Rich.	kpédjrékoun (F)	fruit	T, A, VW	anemia, contraception, infertility, menstruation, postpartum infections, STIs	NC

Botanical Name	Local Name[a]	Used part	Preparation[b]	Use category[c]	AMT#[d]
Zanthoxylum sp.	heja (M)	leaves	T	intestinal cleanse	NC
Zanthoxylum zanthoxyloides (Lam.) Zepern. & Timler	hêdo (F), chanuwele (T)	bark, root, leaves	T, A, E	abortion, anemia, contraception, hemorrhoids- internal, intestinal cleanse, postpartum cleanse, menstruation, pregnancy, postpartum infections, STIs	145, 288, 654
Zapoteca portoricensis (Jacq.) H.M.Hern.	akanmoun (F)	root	T, A	abortion, contraception	NC
Zea mays L.	gbade (F)	flower stigmas (silk), fruit	T, D, E	anemia, galactagogue, cysts, fibroids	NC
Zingiber officinale Roscoe	ata (T)	rhizome	A, T	cysts, fibroids, hemorrhoids- internal	NC

[a] Local languages are abbreviated: (F)= Fon; (Fr)= French; (G)= Goun; (M)= Mina; (N)= Nago; (S)= Seto; (T)= Tcha.

[b] Preparations are abbreviated: (A)= soaked in alcohol; (CS)= chew and spit; (D)= drink; (E)= eat; (EA)= external application; (EN) = enema; (HB)= herbal bath; (J)= juice; (M)= massage; (P)= powder; (T)= tea; (TC) = touch contact; (V)= vapor; (VW)= vaginal wash; (W)= waistband

[c] Use category abbreviations are as follows: CBD= cultural bound disease; HBP = high blood pressure; STIs= sexually transmitted infections.

[d] Botanical voucher number and collector initials; NC= not collected.
