# Supplementary material for: Comparing local perspectives on women’s health with statistics on maternal mortality: an ethnobotanical study in Bénin and Gabon
Source: BMC Complement Altern Med. 2014 Mar 28;14:113. doi: 10.1186/1472-6882-14-113 (PMC3986651; doi:10.1186/1472-6882-14-113)
Supplement: Additional file 2 — Species cited in 41 questionnaires in Gabon. Scientific botanical name, name local language(s), used plant part, preparation, use category and collection number. [file 1472-6882-14-113-S2.pdf]

Additional file 2

Species cited in 41 questionnaires in Gabon: scientific botanical name, name local language(s), used plant part, preparation, use category and collection number

Botanical Name	Local Name[a]	Used part	Preparation[b]	Use category[c]	AMT#[d]
Abelmoschus esculentus (L.) Moench	etatam (F), gombo (Fr), dongodongo(B), mibodo (B)	leaves, fruit	EN, VI, E	childbirth, pregnancy	845
Abutilon mauritianum (Jacq.) Medik.	odongi		D or EN	childbirth	NC
Acalypha paniculata Miq.	oekoenkoenakoen (F)	leaves	VI	childbirth, pregnancy	844
Acanthus montanus (Nees) T.Anderson	nvovo (F), pachango (M)	leaves	EN, D, E, T	childbirth, galactagogue, menstruation	1367, 1317
Aframomum citratum (C.Pereira) K.Schum.	azum (F)	leaves, root	EN	menstruation, stomachache before delivery	NC
Aframomum giganteum (Oliv. & D.Hanb.) K.Schum.	obadzom (F)	fruit	SiB	pregnancy	NC
Aframomum sp.	piment indigiene (Fr), ontonou (Ob)	fruit, leaves, stem	S, VI, EN, EA, D	hemorrhoids, pregnancy, stomachache, vaginal cleanse	1152
Afrostyrax cf. sp.	mujumbu	bark	EN, D	infertility, postpartum infections	NC
Ageratum conyzoides (L.) L.	etombijoro (Om), hediki (M)	plant, leaves	VI, D	menstruation, vaginal cleanse	1318
Albizia sp.	evovule sak (F)	leaves, plant	VI, EN	cyst, stomachache, vaginal cleanse	876, 1228
Alchornea cordifolia (Schumach. & Thonn.) Müll.Arg.	bonjay (B, M), nkabi (F), mabonja (S), mabunzi (Ok), mobonzibonzi (Os)	leaves, plant	D, T, VW, VI	anemia, HBP, malaria, STIs, vaginal cleanse	827, 838, 870, 1180, 1186, 1295, 1408
Allium cepa L.	oignon (Fr)	stem	T	HBP	NC
Allium sativum L.	ail (Fr)	stem	T	HBP	NC
Alstonia cf. *boonei* De Wild.	ekoek (F)	bark	D	vermifuge	855
Alstonia congensis Engl.	makouka (B), okouka (M)	bark	T, D	bodyache, galactagogue, menstruation, stomachache	846, 1190
Amaranthus cruentus L.	folon (F)	leaves, plant	EN, E	childbirth, contraception	1240
Amaryllidaceae sp.	molongu (F)	tuber	EN	pregnancy	869
AMT 1277	moutey (B)	bark	D	contraception	1277
Annickia affinis (Exell) Versteegh & Sosef	nfo (F)	bark	D, T, VW, VI	anemia, childbirth, HPB, pregnancy, STIs	NC
Annona cf. *senegalensis* Pers.		root	D	HBP	NC
Annona muricata L.	corossolier (Fr)	leaves, bark	T, EN	HBP, pregnancy	871

Botanical Name	Local Name[a]	Used part	Preparation[b]	Use category[c]	AMT#[d]
Anonidium mannii (Oliv.) Engl. & Diels	ebom (F)	bark	EN	protect fetus, pregnancy	NC
Anthocleista cf. sp.	ayindo (F)	bark	T	HBP	765
Anthocleista vogelii Planch.	monduando (B, M)	bark, leaves	D, E	general good health, HBP, menstruation, STIs	1258
Antrocaryon klaineanum Pierre	angokon (F)	bark	T, EN, VW	anemia, galactagogue, infertility, placenta removal, postpartum cleanse, postpartum infections, pregnancy, STIs, vaginal cleanse	767, 1204
Aoranthe cladantha (K.Schum.) Somers	tchege (B, M), ibanzao (M)	bark	T, D	galactagogue, infertility, menstruation	896, 1230, 1300
Asparagus warneckei (Engl.) Hutch.		leaves, plant	E, EN	vaginal cleanse, menstruation	1230
Asplenium cf. *africanum* Desv.	ayan (F)	plant	SiB	pregnancy	NC
Aucoumea klaineana Pierre	nkomma (F)			disinfectant	823
Baillonella toxisperma Pierre	moabi (F), adzap (F)	bark	D, EN, VW	infertility, menstruation, placenta, postpartum cleanse, postpartum infections, vaginal cleanse, vermifuge	NC
Bambusa vulgaris Schrad	bambou de chine (Fr)	leaves, stem	D, T	HBP	984
Brillantaisia owariensis P.Beauv.	alembetorro (M)	leaves	VI	vaginal cleanse	NC
Caloncoba cf. *welwitschii* (Oliv.) Gilg	miamongon (F)	bark	VI	vaginal cleanse	NC
Canna indica L.	ekonzok (F)	leaves	HB	headache	1233
Capsicum annuum L	petite piment (Fr)	fruit, seeds	EN, T, VW	backache, childbirth, galactagogue, hemorrhoids, postpartum cleanse, postpartum infections, stomachache, vaginal cleanse	NC
Carapa procera DC.	pongabonga (M)	bark	D	contraception	NC
Carica papaya L.	papaya (Fr)	fruit, leaves, root	E, EN, T	galactagogue, contraception, HBP, STIs	NC
Carpolobia alba G.Don	onong (F)	root, leaves	D, E	childbirth, HBP	NC
Cecropia cf. *peltata* L.	parasolier (Fr)	root	D	infertility	NC
Ceiba pentandra (L.) Gaertn.	baobab (Fr)	bark	D	anemia	882
Chromolaena odorata (L.) R.M.King & H.Rob.	langalanga (Ob)	leaves	EA	sores	1178
Cissus aralioides (Welw. ex Baker) Planch.	agondjie (F)	leaves	EN	menstruation	867
Cissus cf. *oreophila* Gilg & M.Brandt		cord, plant, leaves	T	pregnancy, menstruation	1296
Citrus aurantiifolia (Christm.) Swingle	citron (Fr)	fruit	D, T	HBP, malaria, postpartum hemorrhage, pregnancy	NC
Cleistopholis cf. *glauca* Pierre ex Engl. & Diels	nohoney (Ok)	cord	W	pregnancy	NC
Cleistopholis cf. *patens* (Benth.) Engl. & Diels	ohoey (B)	cord	W	pregnancy	1279
Clerodendrum formicarum Gürke		plant	EN	pregnancy	1236

Botanical Name	Local Name[a]	Used part	Preparation[b]	Use category[c]	AMT#[d]
Coelocaryon preussii Warb.		bark	T	anemia	1305
Cola nitida (Vent.) Schott & Endl.	ngwan (F)	seeds, bark	EN	CBD les urines, childbirth	NC
Cola sp.	kola (F)	seed, bark	D, VW, E	childbirth	NC
Combretum aphanopetalum Engl. & Diels	otoelefok (F)	leaves	VI	vaginal cleanse	866
Combretum racemosum P.Beauv.	mosombasomba (Ok)	leaves	VI	vaginal cleanse	NC
Commelina cf. *diffusa* Burm.f.	essang (F)	leaves	VI	vaginal cleanse	1362
Costus afer Ker Gawl.		flower	T	STIs	NC
Costus sp.	myen (F)	plant, leaves	HB, D, EN	anemia, fibroids, cysts, galactagogue, pregnancy, menstruation, postpartum hemorrhage, vaginal cleanse, vermifuge	NC
Cucumeropsis mannii Naudin	inzaka (B, M), concombre traditionnel (Fr)	fruit, seeds	D, E	childbirth, infertility	854
Cyathula prostrata (L.) Blume	diztaztu (Ok), chabanakoko (B)	flower, plant		HBP, vaginal cleanse	1294, 1400
Cylicodiscus gabunensis Harms	edum (F)	bark	D, HB, T	anemia, pregnancy, vermifuge	NC
Cymbopogon citratus (DC.) Stapf	tisane (Fr)	leaves	T	HBP	NC
Cymbopogon sp.	citronelle (Fr)	leaves, root, stem	SB, D, T	HBP, STIs, pregnancy	NC
Dacryodes cf. *edulis* (G.Don) H.J.Lam	osigi (Ob)	leaves	VI	vaginal cleanse	1181
*Daniellia klaine*i A.Chev.	oengay (B)	bark	T	contraception	1268
Desmodium adscendens (Sw.) DC.	obumen zeney (F)	leaves	E	infertility	NC
Dinophora spenneroides Benth.	massessi (B)	leaves	VI	vaginal cleanse	1194
Dioscorea bulbifera L.	ebouba (M)	fruit	EA	abscesses	1312
Dioscoreophyllum volkensii Engl.	tziga (F)	plant	D	postpartum hemorrhage	1249
Drypetes sp.	esop (F)	innerside bark	VI	vaginal cleanse	878
Elaeis guineensis Jacq.	essong (F)	heart, oil, leaves	E, D	contraception, galactagogue, infertility, placenta removal	NC
Emilia coccinea (Sims) G.Don	alonvoe (F)	leaves	D	menstruation	825
Ethulia cf. *conyzoides* L.f.		leaves	VI	postpartum infections	NC
Euphorbia hirta L.	derebelli (Os)	plant	E	childbirth	1411
Ficus cf. *thonningii* Blume	atak (F)	bark	D, EN, HB	galactagogue, infertility, pregnancy	NC
Ficus exasperata Vahl	ako (F)	bark	D	childbirth, pregnancy	848
Ficus mucuso Welw. ex Ficalho	ekoko (F)	leaves, bark	D, T	anemia, backache	826
Flagellaria cf. *guineensis* Schumach.	enganisang (F)	leaves	EN	postpartum hemorrhage	NC
Fleroya ledermannii (K.Krause) Y.F.Deng	tobu (M), epoukou (M)	bark	D	contraception, infertility, menstruation, pregnancy	897
Funtumia africana (Benth.) Stapf	otanda (B)	bark	D, T	galactagogue	1287

Botanical Name	Local Name[a]	Used part	Preparation[b]	Use category[c]	AMT#[d]
Gnetum africanum Welw.	nkoumou (Ob)	leaves	E	pregnancy	824
Gossypium barbadense L.	coton (Fr)	leaves	T, D	HBP, postpartum hemorrhage	NC
Gouania longipetala Hemsl.	musangia (B)	bark	D	vaginal cleanse	1276
Guibourtia tessmannii (Harms) J.Leonard	obaka (B, M), kevasingo (F)	bark	D, T	diabetes, HBP, infertility, postpartum hemorrhage, pregnancy	764
Halopegia azurea (K.Schum.) K.Schum.		leaves	L	pregnancy	NC
Harungana madagascariensis Lam. ex Poir.	atuin (F)	leaves	HB, E, D	HBP, postpartum infections, pregnancy	NC
Heterotis rotundifolia (Sm.) Jacq.-Fél.	ekaso (F)	plant	VI	vaginal cleanse	1248
Heterotis sp.	ekaso (F)	plant	VI	STIs	NC
Hibiscus sp.	lesseille (F)	flower, leaves	D, E, VI	anemia, vaginal cleanse, pregnancy	NC
Hymenocardia acida Tul.	onganana (Ob)	leaves	D	galactagogue	1155
Hymenocardia ulmoides Oliv.	esang (F)	leaves	VI	stomachache, vaginal cleanse	847
Inga edulis Mart.		seeds	VW	fibroids, cysts	NC
Irvingia gabonensis (Aubry-Lecomte ex O'Rorke) Baill.	andofan (F), mangue sauvage (Fr)	fruit, bark	D, EN	galactagogue, infertility, menstruation	1356
Justicia secunda Vahl	fleur rouge (Fr)	plant, leaves	T	anemia	805, 986
Keetia sp.	ratta (F)	bark, leaves	D, VI	infertility, menstruation, vaginal cleanse	766
Lagenaria cf. sp.	calabasse (Fr), londuh (F)	fruit	B	placenta removal	NC
Laggera cf. *alata* (D.Don) Sch.Bip. ex Oliv.	tabac de pygmée (Fr)	leaves	D	fibroids, cysts	NC
Landolphia cf. *owariensis* P.Beauv.	pondzie	leaves	D	anemia, galactagogue, contraception	NC
Lantana camara L.		leaves	T	malaria	1188
Laportea cf. *aestuans* (L.) Chew	terakun (F)	plant	E, VI	childbirth	NC
Laportea cf. *ovalifolia (Schumach. & Thonn.) Chew*	dipakazangoue (Ok)	leaves		contraception	NC
Leea guineense G.Don	mbala (Om)	bark	D	childbirth	NC
Leguminosae sp.		bark	T	menstruation	1304
Lippia adoensis Hochst. ex Walp.	mututu	leaves	T	galactagogue	NC
Lippia cf. *rugosa* A.Chev.	afing (F)	leaves	T	galactagogue	760
Lippia multiflora Moldenke	punya (S), tisane sauvage (Fr)	leaves	T	HBP, galactagogue	1410
Lippia sp.	lewayi (Ob)	leaves	D	menstruation, postpartum cleanse, postpartum hemorrhage	1179
Macaranga spinosa Müll.Arg	asas (F)	leaves	HB, E, SB, VI	pregnancy, menstruation, vaginal cleanse, diarrhea	1192, 1197
Maesopsis eminii Engl.	enkale (F), mongobe (B)	bark	EN, T	infertility, galactagogue	879

Botanical Name	Local Name[a]	Used part	Preparation[b]	Use category[c]	AMT#[d]
Mangifera indica L.	mangue (Fr)	bark, leaves	VW, T	menstruation, postpartum infections, STIs, stomachache	839
Manihot esculenta Crantz	manioc (Fr)	leaves, tuber	E, EN, D	childbirth, galactagogue, postpartum hemorrhage, pregnancy	NC
Mikania chenopodifolia Willd.	madamoiselle (Fr)	plant	SiB	pregnancy	1242
Milicia excelsa (Welw.) C.C.Berg	abang (F)	leaves, bark	D	galactagogue	835, 836, 1368
Millettia cf. *versicolor* Baker	banjanjoko (B)	bark	T	general good health	1306
Momordica charantia L.	mabubulu	leaves	D	contraception	NC
Morinda lucida Benth.	akon (F)	bark	HB, T, EN	HBP, intestinal cleanse, pregnancy	1213
Musa sp.	umbolokon (F)	leaves, fruit, bark	D, E, EN, SB	anemia, backache, childbirth, galactagogue, menstruation, placenta removal, STIs, vaginal cleanse, vermifuge	NC
Musanga cecropioides R.Br. ex Tedlie	mohombo (B)	leaves	D	childbirth	985, 1309
Myrianthus arboreus P.Beauv.	angokon (F)	bark	D, EN	pregnancy, postpartum cleanse, postpartum infections	853, 1073
Nicotiana tabacum L.	taba (F)	leaves	EN	CBD zchaw, pregnancy	NC
Nymphaea lotus L.	otoetoe (F)	leaves	VI	vaginal cleanse	NC
Ocimum americanum L.	sizey (S)	leaves	T, D	galactagogue, placenta, postpartum cleanse	1407, 1142
Ocimum gratissimum L.	messep (F), masipetzipo (Os)	leaves, plant	D, T, VI	galactagogue, malaria, menstruation, pregnancy, vaginal cleanse, vaginal cleanse	1072, 1409, 1412, 1143
Ocimum sp.	dziandzie (P)	leaves	D	fibroids, cysts	NC
Oryza sativa L.	riz (Fr)	seed	E	galactagogue	NC
Passiflora foetida L.	ejeszum (F)	stem, leaves	EN	infertility, vermifuge	849
Pentaclethra macrophylla Benth.	mpandzi (M), ompie (T)	bark, fruit	EN, D, VW, E	pregnancy, stomachache, vaginal cleanse	1077, 1263
Perichasma cf. *laetificata* Miers	enzigue (F)	root	E	pregnancy	NC
Periploca nigrescens Afzel.	alarminson (F)	leaves	VI	vaginal cleanse	NC
Persea americana Mill.	avocat (Fr)	leaves	T, VI	HBP, vaginal cleanse	982
Petersianthus macrocarpus (P.Beauv.) Liben	abing (F)	bark, leaves	L, SiB, D, S, VI	anemia, backache, pregnancy, vaginal cleanse	1220
Phyllanthus sp.	kanguh (F)	plant	VI	vaginal cleanse	1245
Picralima nitida (Stapf) T.Durand & H.Durand	dumavendo (B, M)	fruit, bark	EN, D	fibroids, cysts, malaria, vermifuge, HBP	1250, 1316
Piper umbellatum L.	abomanzan (F)	plant, leaves	SiB, EN, SB	hemorrhoids, infertility, placenta removal, pregnancy, vaginal cleanse	761
Piptadeniastrum africanum (Hook.f.) Brenan	nlouey (F)	leaves, bark	L, EN	CBD mfoes, infertility	1219
Pistia cf. *stratiotes* L.	angoun (F)	plant	E	infertility	NC
Plagiocladus diandrus (Pax) Jean	mbango (B)	leaves	E	anemia, HBP	1260

Botanical Name	Local Name[a]	Used part	Preparation[b]	Use category[c]	AMT#[d]
F.Brunel					
Plagiostyles africana (Müll.Arg.) Prain	eleesula (F)	bark	EN	menstruation, postpartum infections	NC
Plectranthus monostachyus (P.Beauv.) B.J.Pollard	echipo (M)	plant	D	headache	1319
Poga oleosa Pierre	oayko (M)	plant	EA	sores	1314
Portulaca oleracea L.	dikamiya (Ok)	leaves	T	childbirth	1402
Pseudospondias longifolia Engl.	ofoss (F)	bark	D, EN, T	anemia, stomachache	1081
Psidium guajava L.	guave (Fr)	leaves	SB	malaria, vaginal cleanse	NC
Pteridium aquilinum (L.) Kuhn	ebango (B)			childbirth	1274
Pterocarpus soyauxii Taub.	umbel (F), kaolin rouge (Fr)	bark	D, T, SiB	anemia, galactagogue, inferility, placenta removal, pregnancy, STIs	880, 1203
Pycnanthus angolensis (Welw.) Warb.	mitchoko (B)	bark	T	anemia	NC
Quassia cf. *africana* (Baill.) Baill.	icindural (P)	root	T	HBP	763
Rauvolfia vomitoria Afzel.	tchwele (Ob)	bark	SiB	menstruation	1161
Saccharum officinarum L.	canne sucre (Fr)	juice, stem, plant	D, EN, T	fibroids, cysts, HBP, menstruation	829
Santiria cf. *trimera* (Oliv.) Aubrév.	outou (F)	resin, bark	S	CBD zchaw	NC
Sarcocephalus latifolius (Sm.) E.A.Bruce	ondolo (Ob)	fruit, bark	D, EN	galactagogue, stomachache	1156
Scleria boivinii Steud.	zengey (B)	leaves	D	postpartum hemorrhage, stomachache	1307
Scoparia dulcis L.	nzonzo (F), zedsoro (F)	leaves	D	childbirth	1364
Scyphocephalium cf. *ochocoa* Warb.	sogo (F)	bark	E	menstruation	NC
Selaginella myosurus Alston	ehoyi (M), mahoy (B)	leaves	E	pregnancy, HBP	1063, 1198
Senna alata (L.) Roxb.	kinkiliba (F)	leaves	D, T, S	infertility, STIs	1320
Senna occidentalis (L.) Link	ngari (Ob)	leaves	EA, E	stomachache	1184, 1366
Sida acuta Burm.f.	nzisim (F)	leaves, stem, plant	D, EN, W, SiB, VI	childbirth, CBD ona/onyaboom, CBD les urines, pregnancy, postpartum cleanse	828, 1401, 1177, 1406, 1151, 1254, 1237
Solanecio angulatus (Vahl) C.Jeffrey	moyamboa (B)	leaves	D	childbirth	1298
Solanum americanum Mill.	otchango (M)	leaves	E, D	pregnancy	NC
Solanum anguivi Lam.	petit aubergine (Fr), ombororu (Ob)	fruit	D, T	galactagogue, menstruation, postpartum cleanse, postpartum hemorrhage	NC
Solanum lycopersicum Lam.	tomate (Fr)	fruit	D	anemia	NC
Strychnos cf. sp.		bark	SiB	vaginal cleanse	NC
Tabernanthe iboga Baill.	bois sacre (Fr)	root	E	anemia, contraception, HBP	NC
Tetracera alnifolia Willd.	movova/lian-aho (B)		D	STIs	1259

Botanical Name	Local Name[a]	Used part	Preparation[b]	Use category[c]	AMT#[d]
Tetrapleura tetraptera (Schum. & Thonn.) Taub.	tsélé (M)	fruit	T, EN	galactagogue, menstruation, pregnancy	NC
Tetrorchidium didymostemon (Baill.) Pax & K.Hoffm.	zili (F)	bark	D, EN	galactagogue, infertility, menstruation, postpartum infections	875
Theobroma cacao L.	cacaowey (F)	bark, fruit, leaves	D, T	anemia, HBP, postpartum cleanse	872, 1205
Tithonia diversifolia (Hemsl.) A.Gray	magariet (F)	leaves	D	HBP	NC
Treculia cf. *acuminata* Baill.	mpovo (B)	bark	D	fetus strengthener	1262
Treculia erinacea A.Chev.	edzip (F)	bark	T, EN	anemia, infertility	NC
Trichoscypha cf. *bijuga Engl.*	lokouta (B)	leaves	E	HBP	1299
Trichoscypha sp.	aboet (F)	bark	VW	postpartum cleanse	NC
Tristemma cf. *hirtum* P. Beauv.	masessa	leaves	VI	vaginal cleanse	NC
Tristemma littorale Benth.	abillebong (F)	leaves	VI	vaginal cleanse	861, 0873
Tristemma mauritianum J.F. Gmel.		leaves	T	stomachache	1084
Urena lobata L	okon (F)	leaves	D	postpartum hemorrhage	1238
Vernonia amygdalina Delile		leaves		stomachache	1189
Xylopia aethiopica (Dunal) A.Rich.	oha (M)	leaves	E	HBP	1321

[a] Local languages are abbreviated: (B)= Babungu; (F)= Fang;(Fr)= French; (M)= Mitsogo; (Ob)= Obamba; (Ok)= Okande; (Om)= Omiene; (Os)= Ossimba; (S)= Sake; (T)= Teke.

[b] Preparations are abbreviated: (B)= blow into it; (D)= drink; (E)= eat; (EA)= external application, (EN) = enema; (HB)= herbal bath; (L)= lay upon; (S)= scarification; (SB)= steambath; (SiB)= sitbath; (T)= tea; (VW)= vaginal wash; (W)= waistband.

[c] Use category abbreviations are as follows: CBD= cultural bound disease; HBP = high blood pressure; STIs= sexually transmitted infections.

[d] Botanical voucher number and collector initials; NC= not collected.
